# Long-term effects of remote ischaemic preconditioning in high risk patients undergoing cardiac surgery: follow-up of a randomised clinical trial

**DOI:** 10.1186/2197-425X-3-S1-A411

**Published:** 2015-10-01

**Authors:** A Zarbock, J Kellum, H Van Aken, C Schmidt, S Martens, D Görlich, M Meersch

**Affiliations:** University Hospital Münster, Münster, Germany; University of Pittsburgh, Pittsburgh, PA USA

## Introduction

Acute kidney injury (AKI) is a common complication after cardiac surgical procedures and is associated with an increased morbidity and mortality.

## Objective

In a multicenter randomized trial, we found that remote ischemic preconditioning (RIPC) reduced acute kidney injury (AKI) in high risk patients undergoing cardiac surgery. We now report on the effects of RIPC on long-term outcomes.

## Methods

In this follow up of the RenalRIPC trial, we examined the effect of RIPC the composite end-point of all-cause mortality, need for renal replacement therapy, and persistent renal dysfunction at 90 days (MAKE_90_). Secondary outcomes were renal recovery and dialysis dependence in patients with AKI.

## Results

RIPC significantly reduced the occurrence of MAKE_90_ (17/120 (14.2%)) compared to the sham group (30/120 (25.0%); ARR, 10.8%, 95% CI 0.9%-20.8%, P = 0.034). In the 108/240 patients who developed post-surgery AKI (RIPC 45 (37.5%), Sham-RIPC 63 (52.5%)), 2 (5.3%) in the RIPC group and 11 (22.0%) in the Sham-RIPC did not recovery renal function by 90-days; ARR 16.7%, 95% CI, 3.2%-30.2%, P = 0.028) and 1 (2.4%) versus 8 (15.4%) were dialysis dependent; ARR 13.0%, 95% CI, 2.1%-23.8%, P = 0.036). A receiver operating characteristic (ROC) curve analysis for the MAKE_90_ showed best performance for insulin-like growth factor-binding protein 7 (IGFBP7) and tissue inhibitor of metalloproteinases-2 (TIMP-2) ([TIMP-2]·[IGFBP7]) at 4 h (AUC 0.64; 95% CI 0.547-0.736, P = 0.004). The ROC analyses including AKI positive patients showed that [TIMP-2]·[IGFBP7] was predictive at 4h for renal non-recovery (AUC 0.70; 95% CI, 0.587-0.818; P = 0.021) and at 12 h (AUC 0.74; 95% CI, 0.604-0.879; P = 0.006) after cardiopulmonary bypass. The maximum urinary [TIMP-2]·[IGFBP7] demonstrated an AUC of 0.75 (95% CI, 0.640-0.865; P = 0.004). Optimal cut points were determined from the ROC analyses maximizing the Youden Index ([TIMP-2]·[IGFBP7] at 4h = 0.7; [TIMP-2]·[IGFBP7] at 12h = 0.67, [TIMP-2]·[IGFBP7] maximum = 0.86). Sensitivities ranged from 0.50 to 0.55, specificities from 0.75 to 0.82.

## Conclusions

RIPC significantly reduced the 3-month incidence of a composite end point of all-cause mortality, need for renal replacement therapy, and persistent renal dysfunction in high risk patients undergoing cardiac surgery. Furthermore, RIPC enhanced renal recovery in patients with AKI.

## Trial Registration

The trial is registered at http://www.drks.de (Identifier: DRKS00005333).

